# *QuickStats: *Percentage* of Visits by Patients Aged ≥18 Years to Office-Based Physicians^†^ Made by Patients with ≥2 Selected Diagnosed Chronic Conditions,^§^ by Physician Specialty Category and Patient Age Group — National Ambulatory Medical Care Survey, 2015

**DOI:** 10.15585/mmwr.mm6649a9

**Published:** 2017-12-15

**Figure:**
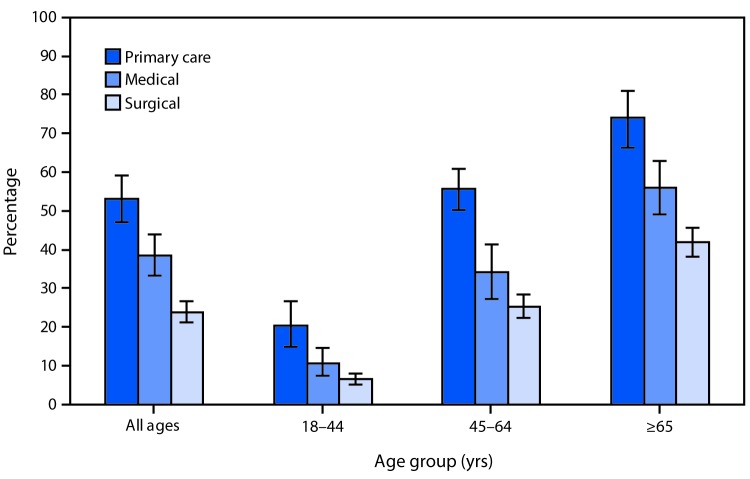
In 2015, the percentage of office-based physician visits by adults with two or more diagnosed chronic conditions was 53.1% for primary care physicians, 38.5% for medical specialists, and 23.9% for surgeons. This pattern was observed for each of the age groups studied. The percentage of visits increased with age group, regardless of specialty category.

**Source:** National Ambulatory Medical Care Survey, 2015 data. https://www.cdc.gov/nchs/ahcd/ahcd_questionnaires.htm.

